# Advancements in self-assembling peptides: Bridging gaps in 3D cell culture and electronic device fabrication

**DOI:** 10.1177/08853282241240139

**Published:** 2024-03-19

**Authors:** Azadeh Jafari

**Affiliations:** Faculty of Applied Sciences, 120447 Simon Fraser University, Surrey, BC, Canada

**Keywords:** Self-assembling peptides, 3D cell culture, biomimetic microenvironments, tissue engineering, neuro-supportive

## Abstract

Self-assembling peptides (SAPs) show promise in creating synthetic microenvironments that regulate cellular function and tissue repair. Also, the precise π-π interactions and hydrogen bonding within self-assembled peptide structures enable the creation of quantum confined structures, leading to reduced band gaps and the emergence of semiconductor properties within the superstructures. This review emphasizes the need for standardized 3D cell culture methods and electronic devices based on SAPs for monitoring cell communication and controlling cell surface morphology. Additionally, the gap in understanding the relationship between SAP peptide sequences and nanostructures is highlighted, underscoring the importance of optimizing peptide deposition parameters, which affect charge transport and bioactivity due to varying morphologies. The potential of peptide nanofibers as extracellular matrix mimics and the introduction of the zone casting method for improved film deposition are discussed within this review, aiming to bridge knowledge gaps and offer insights into fields like tissue engineering and materials science, with the potential for groundbreaking applications at the interface of biology and materials engineering.

## Introduction

Short sequences of amino acids connected by peptide bonds are called peptides. The building components of peptides and proteins are amino acids. Between the early 19th and the first part of the 20th centuries the first amino acids were identified.^1^ Proteins are made up of 50 amino acids or more, whereas peptides are often considered as entities with between 2 and 50 amino acids.

Peptides and their derivatives can self-assemble to produce a variety of useful nanomaterials, notably for use in biomedical and optoelectronic applications. Nanofiber-forming peptides are intriguing as scaffolds for tissue engineering because they may simulate key aspects of the extracellular matrix (ECM), such as its fibrous morphology, mechanical characteristics, and chemical cues.

Topographical and biological cues play crucial roles in the ECM in directing tissue orientation and function. As a result, many tissues, including muscle, tendons, ligaments, and nerve tissue, are made up of macroscopically aligned fibers that control the qualities of the tissue. In order to regenerate tissue, biomimetic scaffolds that can mimic aligned structures may be necessary.

The process of molecular self-assembly is a reversible and spontaneous organization of some molecular entities (small or macromolecules, peptides, or proteins) that was generated under thermodynamic equilibrium into a well-defined and stable arrangement without the use of outside assistance.^1^ A unique family of self-assembling and self-recognition molecules, peptides and, more recently, peptidomimetics, can function as organic semiconductors with applications in basic biology and healthcare. Due to their environmental, morphological and functional flexibility, and ease of preparation, modification, and doping, self-assembling peptide nanostructures may provide an alternative supply for the semiconductor industry. Moreover, the diverse bottom-up methodologies of peptide self-assembly facilitate easy and cost-effective device fabrication, with the ability to integrate external functional moieties. For instance, n-p junctions may be built using the assembly of peptides and electron donors or acceptors, and custom electronics and chips can be produced on a variety of substrates using vapor deposition techniques. Self-assembling peptide research has been going on since the 1990s.^2^ In order to maintain peptide-based self-assembled structures in a stable low-energy state, hydrogen bonding, hydrophobic interactions, electrostatic interactions, and van der Waals forces combine.^3^ Several factors such as amino acid sequence, the degree of hydrophobicity, the length of the peptides, and the self-assembling time affected on the self-assembling processes. Moreover, in self-assembling peptides (SAPs), when multiple assemblies are put together, a supramolecular network can be established biocompatibility. Also, SAPs can be used for intracellular or targeted tissue delivery of various nucleotides and antibodies for therapy, and for the delivery of drugs that cannot be easily mobilized owing to their physicochemical characteristics or those that exhibit a rapid clearance rate. On the other hand, they can be applied to the treatment of various diseases as peptide drugs. Also, by pH, ionic strength, temperature, or enzymatic triggers the self-assembly of peptides can be controlled.^4^ Self-assembling peptides (SAPs) come in various typologies, each with its unique properties and potential applications. Here are some common types:

### β-sheet forming peptides

These peptides typically self-assemble into β-sheet secondary structures, forming nanofibers or nanotubes. The β-sheet structure is known for its stability and rigidity, making it suitable for applications requiring mechanical strength, such as tissue engineering scaffolds.^5^

### α-helical peptides

In contrast to β-sheet peptides, α-helical peptides form α-helix secondary structures. These peptides may assemble into different nanostructures such as helical bundles or coiled-coil structures. They are often employed in applications where flexibility and dynamic interactions are desired.^6^

### Amphiphilic peptides

Amphiphilic peptides contain both hydrophobic and hydrophilic regions within their sequence. This property allows them to self-assemble into micelles, vesicles, or bilayer structures, mimicking cell membranes. They are utilized in drug delivery systems, membrane protein stabilization, and as models for studying membrane biophysics.^7,8^

### Hydrogelating peptides

Some peptides have the ability to form hydrogels through self-assembly, resulting in three-dimensional networks capable of retaining large amounts of water. These hydrogels find applications in tissue engineering, wound healing, and drug delivery due to their biocompatibility and similarity to the extracellular matrix.

### Cyclic peptides

Cyclic peptides form closed loops instead of linear chains. Their cyclic structure enhances stability and often confers specific binding properties. Cyclic SAPs are investigated for applications such as molecular recognition, enzyme inhibition, and drug design.^9–14^

### Designer peptides

With advancements in peptide design and synthesis techniques, researchers can now engineer peptides with specific functionalities tailored to particular applications. Designer peptides may combine elements from different typologies to achieve desired properties, such as enhanced biocompatibility, targeting capabilities, or enzymatic activity.

These typologies represent just a few examples of the diverse range of SAPs available for various biomedical, materials science, and biotechnological applications. Each type offers unique advantages and challenges, and ongoing research continues to expand the repertoire of self-assembling peptide-based materials.^14–19^

Over the past 10 years, interest in biologically active materials especially peptides that can be used as cell culture substrates for medicinal applications has increased dramatically. For instance, the shape, size, and type of the inter-chromophore π- π interactions, which are determined by the assembly processes, affect the characteristics of optoelectronic materials.

SAPs, with nanofibrous networks mimicking extracellular matrix (ECM), have shown a remarkable potential because of their synthetic source, biocompatibility and biomimetic properties.^5^ In order to promote neurite outgrowth, neuron differentiation and cell adhesion, SAPs can be customized by adding functional motifs, functionalized or multifunctionalized for different applications and therapeutic treatments. For this reason, recently engineering of nanostructures formed by self-assembling peptides can work as a scaffold in vivo. Because of biodegradability and biocompatibility of peptides, they are excellent candidates for the development of 2D and 3D cell-culture materials and make it possible to tailor biomaterials for different applications in tissue engineering and regenerative medicine. It is unlikely that peptides will passively diffuse across the cell membrane, but altering their physical properties (such as conformational flexibility and polarity) has been proposed to improve their permeability. Nerve tissue is a biological molecule related to the function and maintenance of normal nervous tissue. An example would include, for example, the generation of myelin which insulates and protects nerves.

In this regard, whereas 2D scaffolds present highly sensitive ligands bound to an inert substrate, elasticity and stiffness are key in obtaining differentiation signals for stem cells embedded in 3D scaffolds. In addition, owing to the characteristics of neuronal cells, the regeneration or rehabilitation of neuronal tissue is difficult because lower organisms possess an extensive capacity for neural regeneration, higher organisms, including humans, have limited ability to regenerate nerve cells.^6^ There are several examples of using self-assembling peptides in neuronal cell regeneration:

RADA16-I, composed of natural amino acids, is a kind of injectable self-assembled polypeptide which can form a hydrogel scaffold with a large number of nanofibers under certain conditions. The results based on RADA16-I and II as scaffolds in immature mouse cerebellum and rat hippocampus cells has been showed successful extensive neuron outgrowth. This growth was sustained for more than 4 weeks and resulted in the formation of an active synaptic connection. Also, it has been shown that RADA16 could be used to support neural stem cells and primary neurons. The self-assembling peptide nanofiber scaffold composed of RADA16 resulted in reconnection of the injured spinal cord and facilitated axon regeneration and, eventually, locomotor functional recovery in animal models of spinal cord injury.^7,8^

Another example of using a self-assembling peptides in neuronal cell regeneration is the -IKVAV penta-peptide combined with -Glu- and A4G3-alkyl residues. It has been shown that the -IKVAV- peptide can be used to promote neurite sprouting and growth. Also, they can increase the fibril formation of self-assembled structures, improved cell growth, promoted neuronal cell differentiation, and inhibited astrocyte differentiation.^20^ Also, it has been shown that –SKPPGTSS, -PFSSTKT-, and RGD motifs combined with the RADA16-1 peptide increased the levels of nestin, β-tubulin, and other neuronal markers.^9^

In addition, it has been shown that the combination of the self-assembling peptide QL6 with neural precursor cells (NPCs) neural progenitor stem cells enhanced neuro-behavioral recovery, increased neuronal conduction, and improved survival.^21^ Nerve injury was induced by clip compression of the rodent spinal cord. SAPs were injected immediately into the injured cord and NPCs at 2 weeks post-injury. QL6 enhanced neuronal differentiation and axonal regeneration and suppressed astrocytic development with a reduction in post-traumatic apoptosis, inflammation, and astrogliosis.^22^ Beside nanofibrous scaffolds, amyloids represent promising peptide species as substrates to control stem cell behavior. A series of peptides to form amyloid nanofibril based hydrogels for 2D and 3D stem cell culture and differentiation have been developed. These amyloid nanofibrils, consisting of self-assembling Fmoc-protected peptides derived from β-sheet prone C-terminal Aβ42 are non-toxic, thermo-reversible and thixotropic. For salts dependent study, peptides were dissolved in presence and absence of NaCl and the gels were prepared as described previously.^23^ By varying the peptide and salt concentration, the stiffness of the resulting amyloid gels can be modulated. Most of them are supporting cell attachment, proliferation and influence the stem cell fate.^23^

The nanoscale ECM manipulation changes cell growth’s functional and mechanical properties. In order to simplify and control of the 3D cell culturing process the 2D cell culture is proposed on a well-defined substrate with drastically different mechanical and biochemical properties. For controlled 2D culturing a plethora of natural and synthetic polymers, recombinant proteins, ceramics, and metal-composite scaffolds are proposed. The structurally highly organized materials like self-assembled peptides (SAPs) and nanomaterials (NMs) on a macroscopic scale can simulate a natural ECM. Self-assembling peptides and peptide conjugates have high potential capability due to their biocompatibility, morphology, biodegradability, and bio-functionality. Understanding and controlling the peptide self-assembly process may help create more functional nanostructures. One of the main advantages is that the properties of SAPs can be controlled and scaled by temperature, pH, concentration, and nature of ions in the solution.^8,9,20^

It can be summarized, that the nucleation and growth mechanism during the self-assembly process into corresponding structures is governed by the above described intermolecular interactions as well as conditions between different series of the amino acids forming a single peptide’s structure. Such peptides can self-assemble into diverse nanostructures such as nanofibers, nanotubes, nanoarrays etc.^10–12^ These processes happen under thermodynamic and kinetic requirements resulting from specific and local molecular interchanges. Hydrogen bonding, hydrophobic and electrostatic exchanges, and van der Waals energies connect to keep molecules at a stable low-energy state. Self-association to create hierarchical networks at both the nano and microscales happens to accomplish these energy minima.^13,14^ The long-range order, homogeneity and continuity of the bimolecular scaffold determine the quality of the cell cultured films. From this reason, self-assembling peptides with well-defined surface morphology are promising scaffolds for cellular growth, adhesion, proliferation, and migration. Regardless of the created form, they exhibit properties such as biocompatibility and molecular recognition and, more importantly, superb resistance to extreme conditions of detergents and denaturants, and a wide range of temperatures.^10–14^ A significant asset of self-assembling peptides is the ability to build nanostructures in a bottom-up approach. This is equivalent to that these structures are built from the self-integration of small, simple building blocks. Mostly, this approach is required for the nanoscale structure because the top-down techniques are not useful in biological applications.^15,16^ Formation of defined structure like β -sheet or α-helix of peptide in one direction on large area are one of the major requirement for creation of cell scaffold. However, lack of understanding and controlling the peptide self-assembly process into macroscopically aligned fibers for cell culturing reduces their possible potential application as biologically active 2D extracellular matrix.

Overall, this work contributes to advancing the understanding and application of self-assembling peptides in tissue engineering, biomaterials science, and electronics by addressing challenges related to standardized methodology, morphological control, and film deposition techniques.

This review presents a novel approach utilizing self-assembling peptides (SAPs) to create biomimetic micro-environments for tissue engineering and electronic device fabrication. Addressing the challenge of reproducibility and tenability in 3D cultures, the study highlights the importance of understanding the mechanistic relation between peptide sequences in SAPs and resulting nanostructures. By optimizing peptide deposition parameters and employing the zone-casting method, the research achieves improved control over the morphology and organization of peptide nanofiber networks. This advancement not only enhances the structural integrity and functionality of tissue scaffolds but also facilitates the development of electronic devices capable of monitoring cell-cell communication. The integration of zone-casting techniques with the deposition of organic semiconductors offers a promising avenue for producing well-defined surface morphologies of biomolecules, thus advancing both tissue engineering and electronic device fabrication fields.

## The most widely used methods for peptide deposition

Peptides are chemically synthesized by the condensation reaction of the carboxyl group of one amino acid to the amino group of another one. Selection of protecting groups for amino acids and deprotection in peptide bond formation are two basic chemical principles in peptide synthesis. In order to peptide production, two chemical techniques have been developed: (1) solid phase peptide synthesis (SPPS) and (2) solution phase synthesis (SPS):

SPPS is a common technique for peptide synthesis. Usually, peptides are synthesised from the carbonyl group side (C-terminus) to amino group side (N-terminus) of the amino acid chain in the SPPS method, although peptides are biologically synthesised in the opposite direction in cells. This method allows the rapid assembly of a peptide chain through successive reactions of amino acid derivatives on an insoluble porous support.

**Figure 1.**
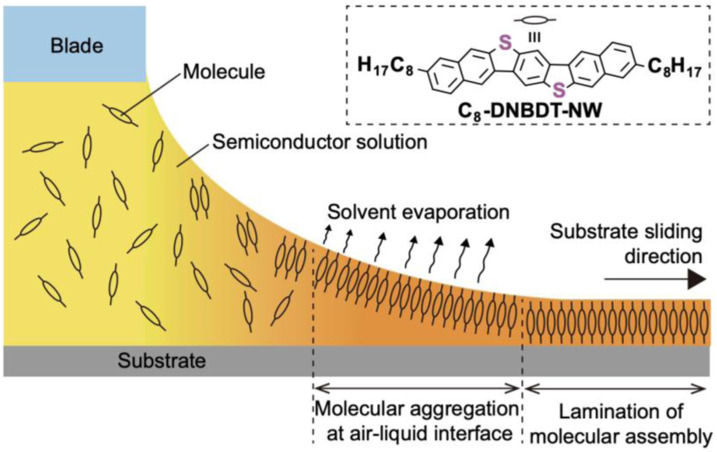
Schematic image of meniscus-guided coating.^26^

On the other hand, SPS is based on the coupling of single amino acids in solution. The prime advantage of SPS for peptide synthesis is that the intermediate products can be deprotected and purified to give the final desired peptide in high purity. Although SPS can be scaled up in an easy manner, the long reaction time remains a disadvantage of this process. Oxytocin, porcine gastrin releasing peptide and human insulin are a few examples of peptide hormones that were synthesized by SPS.^24,25^ Meniscus-guided coating (MGC) techniques are widely recognized as a simple, efficient, and low-cost methods to achieve cost-effective high-throughput and large-area organic electronics as well as peptides. One of the major challenges is the control of thin film morphology, molecular orientations and directional alignment of polymer films during coating processes. Figure 1 show schematic image of meniscus-guided coating technique. Solvent evaporation at the air–liquid interface induces a pre-aggregation of solute molecules, forming crystal nucleation. The soft landing of the nuclei subsequently initiates crystallization.^26^

Shearing speed, solute concentration, deposition temperature, and solvent boiling point are factors that important to analyze crystal growth behavior in the meniscus‐guided coating. For instance, high boiling point solvent corresponds to a lower crystallization rate, which allows the molecules enough time to self-organize, and resulting in larger crystal domains. In fact, when high boiling point solvents were used in MGC techniques, the substrate needs to be heated up to raise the deposition efficiency of OSC thin films. Common MGC methods include dip coating, zone casting, spin coating and drop casting.

### Dip coating

**Figure 2.**
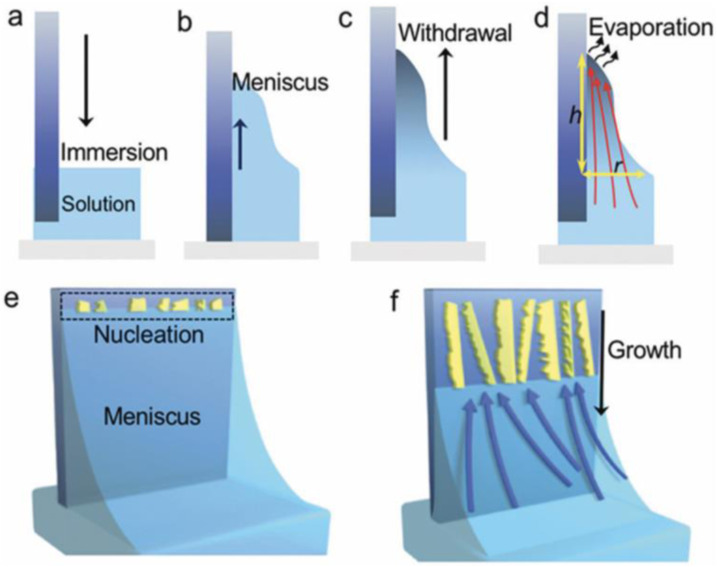
Main stages in dip coating process.^27^

In dip coating technique, thin films using a purpose-built dip coater is formed. In this method, highly uniform films formed under optimal conditions. Importantly, key factors such as film thickness can be easily controlled. In dip coating method, the substrate is immersed in a liquid and then withdrawn with a well-defined withdrawal speed under controlled temperature and atmospheric conditions. Dip coating process include 4 stages: immersion, dwelling, withdrawal and evaporation (see Figure 2).^27^

The advantages of dip coating are that dip coating provides a protective shield that resists corrosion, insulates against heat, cold, stress and electrical currents, adaptable to high volume orders requiring fast delivery, durable and UV resistant.

A general direct synthesis strategy (straightforward method), yielding hybrid bioorganic–inorganic materials based on peptides and silica has been reported. In this study, instead of relying on surface modification chemistry to immobilize a bioorganic moiety on silica, protected hybrid peptides selectively functionalized with a trialkoxysilyl group at a suitable position has been synthesized. Also using dip-coating method peptides on piezoelectric crystals surface has been coated. Peptide (LEKKKKDC-NH2) derived from an aldehyde binding site in the HarmOBP7 protein was synthesized and used as a sensing material for the biosensor.^28^

### Zone casting

**Figure 3.**
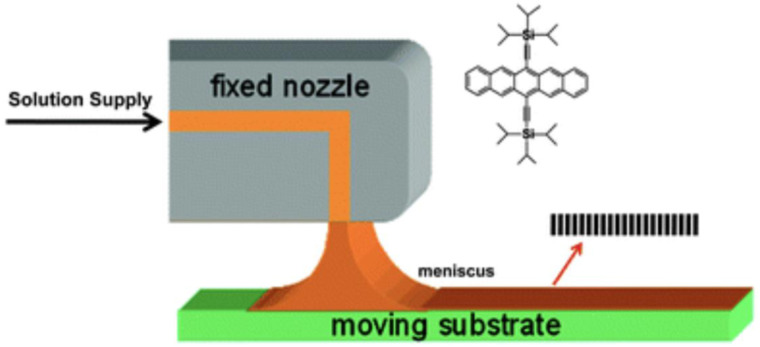
Schematic representation of the process of zone-casting technique.^27^

Zone-casting technique is a method for the preparation of oriented, anisotropic layers of soluble molecular materials on substrates which solution is supplied onto a moving substrate through a flat nozzle, and a meniscus will form between the flat nozzle and the substrate (see Figure 3). Zone-casting technique can provide a stable solution flow rate, which can determine the resulting thin film thickness. In fact, it is a method to align discotic liquid crystal molecules, oriented, anisotropic layers of soluble molecular materials on substrates which possess a large overlap of π orbitals between neighboring molecules. Several factors influence on material property on substrate: solution supply rate, substrate velocity, initial solute concentration, solvent evaporation rate. The key of this technique is to define the ordered domain nucleation at the solidified front and to control its advancing rate through the solvent evaporation.^27^

### Spin-coating

**Figure 4.**
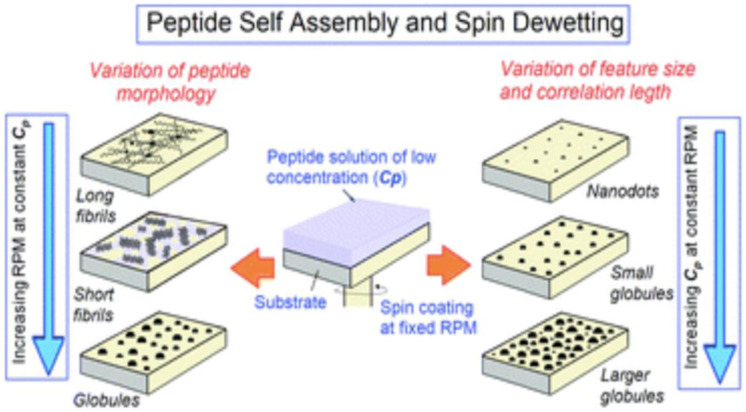
Varying the spinning speed (RPM) and the solution concentration (*C*p) during spin coating.^29^

Spin coating is a technique to spread uniform thin films by a spin coater on flat substrates at high speed using centrifugal force. In this method, the high angular velocity spreads the solution over the substrate surface and leads to a high solvent evaporation rate. The thickness of spin-coated films is inversely related to spin-coating speed and depends on the solution concentration and viscosity. Factors such as final rotational speed, acceleration, and fume exhaust contribute to how the properties of coated films are defined. The final film thickness will be inversely proportional to the spin speed and spin time. Final thickness will be proportional to the exhaust volume although uniformity will suffer if the exhaust flow is too high since turbulence will cause nonuniform drying of the film during the spin process. It has been shown that spin coating, has been used to modulate the self-assembly of peptide molecules, which has traditionally been achieved by chemical functionalization of the molecules. With the specific example of diphenylalanine-based peptide molecules, it has been shown that a variety of self-assembled architectures such as long fibrils, short fibrils, globules, nanodots, and so on, spanning over large areas can be obtained by simultaneously varying the spinning speed (RPM) and the solution concentration (*C*p) during spin coating (see Figure 4).^27,29^

**Figure 5.**
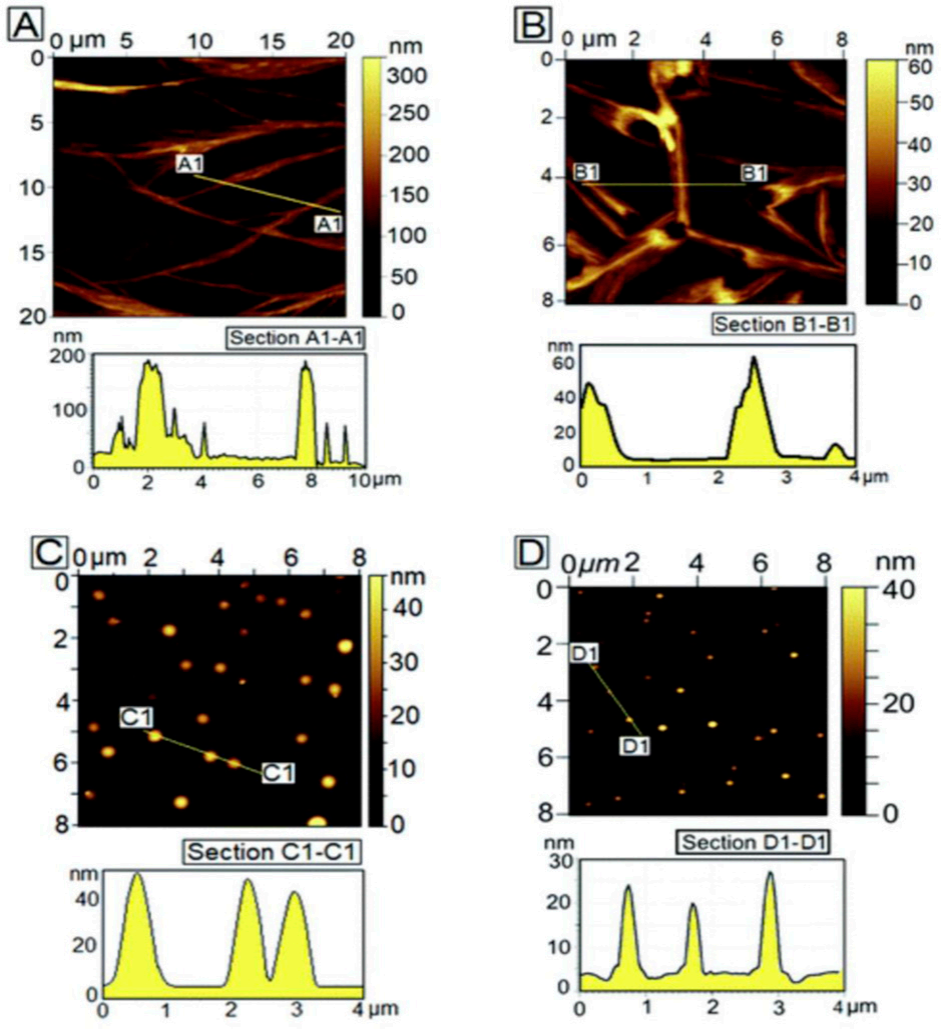
AFM images of Boc-FF-OMe on a glass substrate as a function of RPM: (A) Drop casting of the solution with slow drying at room temperature results in long fibrils with random distribution. (B) 500 r/min, short fibrils uniformly distributed over the substrate. (C) 1500 r/min, peptide globules. (D) 2500 r/min, peptide globules of smaller size.

In this research spin coating for obtaining uniform assemblies of Boc-FF-OMe (N-tert-butyloxycarbonyl-di-l-phenylalanine methyl ester) and three other variants of FF peptides spanning over large (m^2^) areas. Figure 5, shows the progressive change in the self-assembled morphology of Boc-FF-OMe on a glass substrate as a function of RPM: AFM images of Boc-FF-OMe peptide assemblies obtained from spin-coating a solution with solution concentration *C*p = 1 mg mL^−1^ at different spinning speeds. The AFM image in Figure 5(A) was obtained around the centre of the dispensed droplet, where the morphology was relatively uniform. Consequently, at lower RPM, a higher number of peptide molecules remain available after splashing in comparison to a greater number of molecules being splashed out at higher RPM. The distinction in morphologies in Figure 5(B) (500 r/min), 6C (1500 r/min), and 6D (2500 r/min) is the clear signature of the above statement. Further, with the increase in RPM as a lower number of peptide molecules are available at 1500 r/min and 2500 r/min, they fail to assemble into fibers and result in the formation of isolated peptide globules (Figure 5(C) and (D)).

### Drop-casting

Drop-cast is the formation of a thin solid film by dropping a solution onto a flat surface followed by evaporation of the solution. In this method, an evaporation take place at the substrate-solution contact line, therefore the nucleation and self-assembly starts from the contact line and gradually moves to the center region of the drop or substrate. Its advantages over spin-coating include less solution wastage and sufficient time scale for solution self-assembly.^27^ Therefore, drop-cast films generally show better charge transport as compared to spin-coated films due to the improved molecular organization, induced by a slower growth processes.

**Figure 6.**
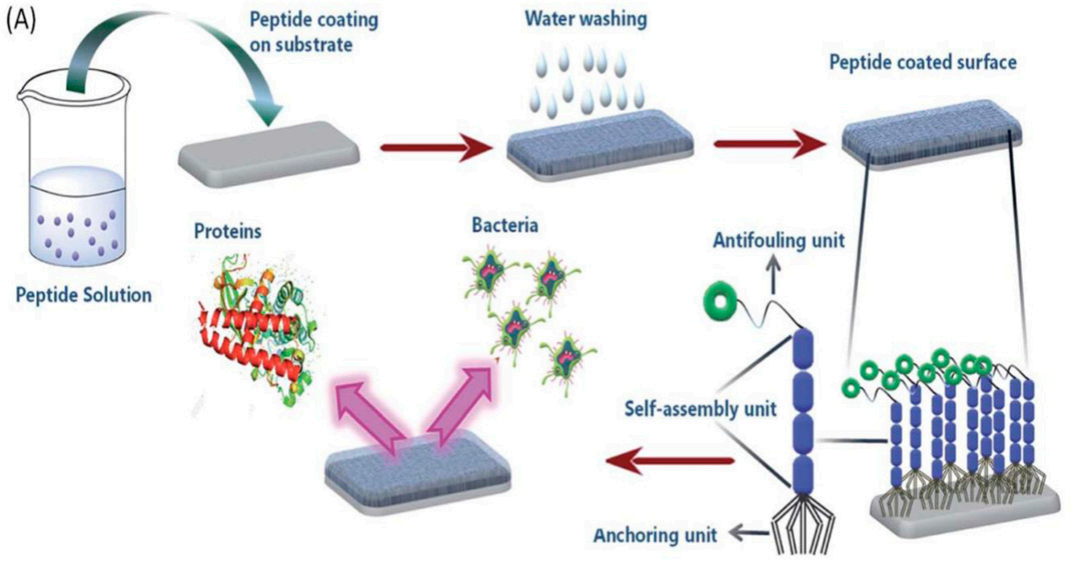
Formation of antifouling coating based on the self-assembly of peptides.^30^

Recently, the design and synthesis of two simple chemically modified peptides, namely, PA1 (PFB-VVD) and PA2 (PFB-LLE) by drop casting method has been reported.^19^ Both the peptides form functional supramolecular coatings on the silica surface that are able to resist biofilm formation. Figure 6 shows the formation of antifouling coating based on the self-assembly of peptides. This figure represents the formation of a supramolecular coating on the desired substrate by the self-assembled tripeptides (PA1, PA2, having three different units; anchoring unit, self-assembly unit and antifouling moiety) exhibiting antifouling activity.^30^

**Figure 7.**
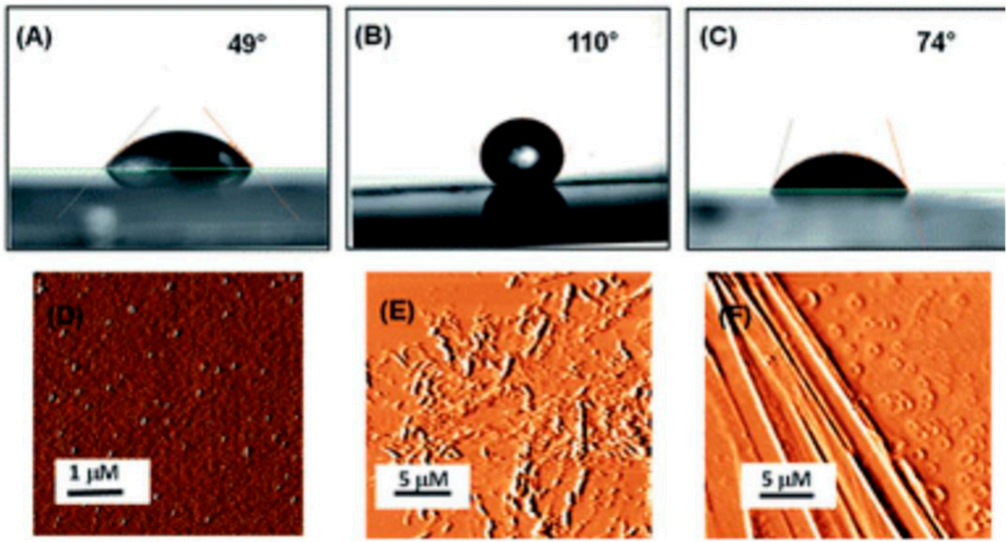
Contact angle measurements of (A) uncoated silica, (B) coated with peptide PA1, (C) coated with peptide PA2 and AFM topography images of (D) uncoated silica, (E) coated with peptide PA1, (F) coated with peptide PA2.^30^

Surface characterization and contact angle measurements of bare silica surface and silica surface coated with peptides PA1 and PA2 has been shown in Figure 7. AFM analysis clearly revealed that there is a substantial difference in the topography of the peptide-coated silica surfaces and bare silica surface. In addition, some self-assembled supramolecular aggregates of the respective peptides appeared on the coated substrate. Understanding the interaction of the peptide-coated surfaces with water is essential as both hydrophobicity and hydrophilicity play a significant role in the design of smart and efficient antifouling materials.^31^ It was observed that the hydrophobic nature of the peptide-coated surfaces was increased compare to that of the bare surface. The increased hydrophobicity of the Teflon-like layer coated surfaces was confirmed by contact angle measurements with water droplets. The results showed an increase in the water contact angle from 49° to 110° (with the PA1 coated surface) and from 49° to 74° (with the PA2 coated surface) (Figure 7(A)–(C)). AFM analysis clearly revealed that there is a substantial difference in the topography of the peptide-coated silica surfaces and bare silica surface.^30^

## Application of peptide in cell culture scaffolds

**Figure 8.**
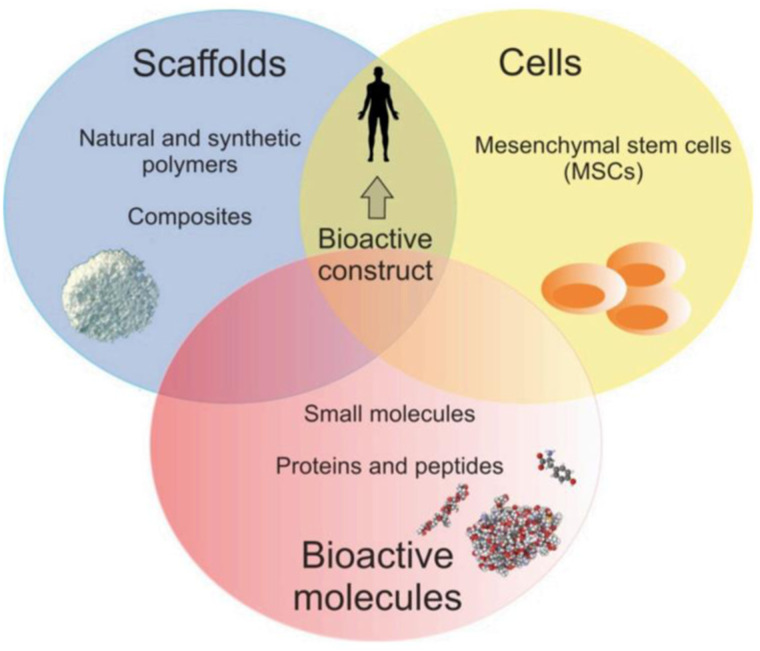
The classical TE paradigm including scaffolds, cells, and bioactive molecules.^32^

Tissue engineering (TE) is a biomedical engineering discipline, which constitutes an alternative and promising approach for grafts, combining scaffolds, cells, and biologically active molecules into functional tissues. The main assumptions of the TE are presented in Figure 8. It was found that peptide-modified biomaterials exhibit structural and biological properties close to those of protein-modified scaffolds and peptide-based biomaterials are in many instances capable of mimicking the structure and function of their full-length endogenous counterparts. In fact, production of peptides is simpler and more cost-effective compared to the fabrication process of full-length and high-molecular weight proteins. In addition, peptides are more resistant to environment conditions (e.g., pH and temperature) and they are relatively safe due to a low immunogenicity.^32,33^

In the classical TE strategy, suitable scaffolds play an important role and they are three-dimensional (3D) matrices, which mimic native extracellular matrix (ECM) to support cell growth. Scaffolds provide the structural support for cell attachment and should be biocompatible, biodegradable at the desired rate, porous, and mechanically stable. The ECM is composed of adhesive glycoproteins, proteoglycans, fibrous proteins and glycosaminoglycans (GAGs). In addition, growth factors also occur within ECM and play an important role as cellular modulators. Thus, improvement of polymer scaffolds with proteins and peptides enables fabrication of biocompatible biomaterials that mimic natural ECM.^33^

### Peptide nanofiber scaffold

**Figure 9.**
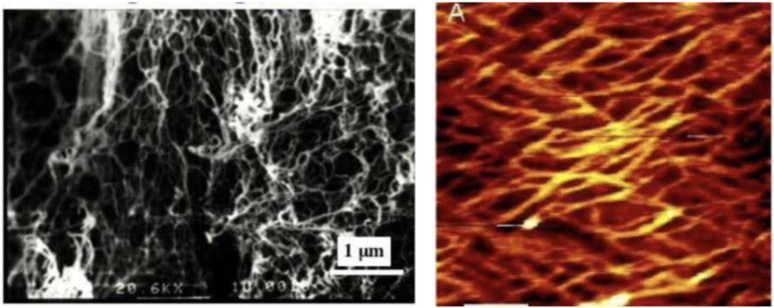
SEM and AFM images of EAK16-II nanofibers.^34^

FEAK16-II is the first member in the self-assembling peptide family and an EAK membrane-like scaffold was first discovered in the tissue culture media where PC12 cells were used to test for EAK16-II cytotoxicity. EAK16-II nanofiber formation was demonstrated using SEM and AFM. The nanofibers are ∼10–20 nm in diameter with remarkable structural regularity (see Figure 9). The PEAK scaffold showed no apparent toxicity, the PC12 cells were found to attach onto the membranous materials.^34^

**Figure 10.**
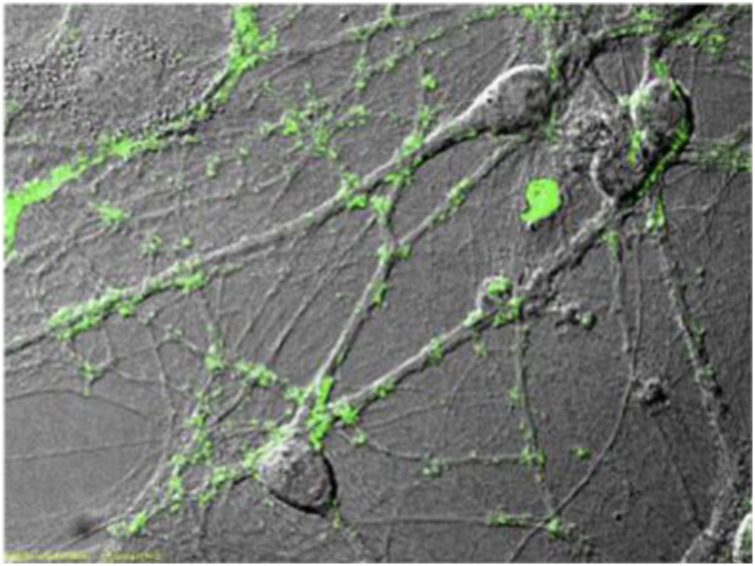
Active synapses on the peptide surface. Primary rat hippocampal neurons form active synapses on peptide scaffolds. The confocal images shown bright discrete green dot labeling indicative of synaptically active membranes after incubation of neurons with the fluorescent lipophilic probe FM-143.^35^

Pura Matrix Peptide Hydrogel is a synthetic matrix used to create defined three dimensional (3D) micro-environments for a variety of cell culture experiments. Pura Matrix not only can be used as a coating or to encapsulate cells similar to the ECM, but can also be tailor-made for particular cells, tissues and therapies. PuraMatrix act as a substrate and portable membrane media to support both neuronal attachment and differentiation in the form of extensive neurite outgrowth. In Figure 10, active synapses on the peptide surface has been shown. Primary rat hippocampal neurons form active synapses on peptide scaffolds. The active synapses on the peptide scaffold are fully functional, indicating that the PuraMatrix is a permissible substrate for neurite outgrowth and active synapse formation.^35^

### Laminin peptide-conjugated agarose micro-gels as a cell culture scaffolds

**Figure 11.**
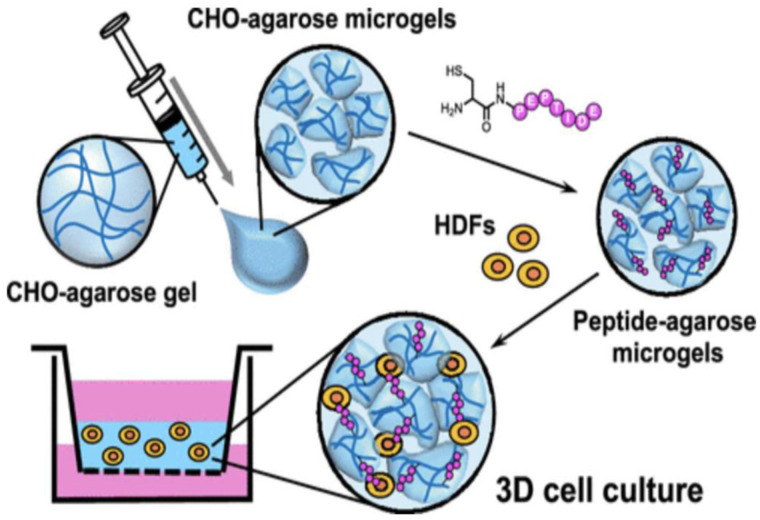
Preparation of the peptide-agarose microgel scaffold for three-dimensional (3D) cell culture.^36^

A method for preparation of functional 3D cell scaffolds using laminin-derived cell-adhesive peptides and agarose has been reported. Laminins as high-molecular weight protein consist of three peptide chains: α, β, and γ and. Laminins, the first ECM glycoproteins detectable in the embryo, are found in basement membrane. Various polymer hydrogels have been utilized to design 3D scaffolds as a biomaterial. Agarose is a polysaccharide derivative of agar. Also, agarose gels need to be combined with cell adhesive biomolecules, such as proteins and peptides, when used for culture of anchorage-dependent cells. Figure 11 depicts a schematic illustration of the preparation of the peptide-agarose microgels and 3D cell culture using the micro-gel assembly scaffold.^36^

**Figure 12.**
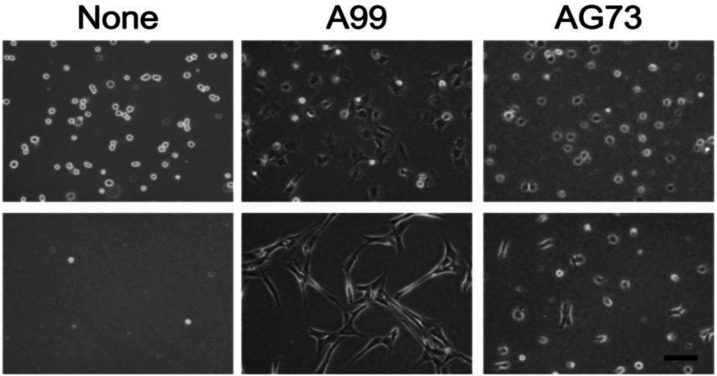
HDFs cultured on control (None), A99-, and AG73-agarose gels under serum-free conditions for 1 and 24 h (top and bottom). Scale bar = 100 μm.^36^

In this study, two-dimensional (2D) cell culture assay using human dermal fibroblasts (HDFs) showed that the peptide-agarose gels have potent cell adhesion activity and promoted cell proliferation. HDF morphology on the gels in the absence of serum has been shown in Figure 12. 1 h after seeding, the cells on the surface of the control agarose gel (None) had a spherical shape and then the cells disappeared from the gel surface, indicating that the agarose gel itself did not have cell adhesiveness, as expected. Meanwhile, HDFs strongly attached to the surface of the A99-and AG73-agarose gels and remained attached to the gels over 24 h. The attached cells on the A99-agarose gel showed extensively elongated morphology, which was typical of integrin-mediated adhesion of fibroblasts. The cells attached to the AG73-agarose gel did not spread extensively, even after 24 h. It has been reported that the A99-mixed agarose gel does not have cell attachment activity and the AG73-mixed agarose gel promotes cell adhesion but maintains the attached cells only for the short term.^36^

## Application of peptide to support neural stem cells and primary neurons

Numerous cell types require interactions with extracellular matrix (ECM) molecules. These cell types include stem cells, primary epithelial cells, and primary endothelial cells. There are many problems related to their use of animal- and human-derived ECM molecules, even though they are necessary for cell culture applications. Arg-Gly-Asp (RGD); Ile-Lys-Val-Ala-Val (IKVAV); Tyr-Ile-Gly-Ser-Arg (YIGSR); Arg-Tyr-Val-Val-Leu-Pro-Arg, or RYVVLPR; Arg-Asn-Ile-Ala-Glu-Ile-Ile-Lys-Asp-Ile, or RNIAEIIKDI are short peptides which have been identified from full ECM molecules to support cell adhesion and growth. The advantages of short peptide sequences compared to whole proteins are: more stable, more easily synthesized and are less likely to exhibit steric hindrance.^37^ Although many short peptide sequences have been identified, none of these peptides from commercial sources can support attachment, growth, and long-term culture of human neural stem/progenitor cells (hNSCs).

### Peptide nanofiber for culturing of primary neurons

Networks resembling the extracellular matrix in key aspects that could serve as an artificial scaffold structure promoting cell adhesion and growth, can form from peptide nanofibers (PNFs). In recent research, self-assembly peptide nanofibers with alternating polar residues were employed for biological applications, such as tissue engineering scaffolds, drug release matrices and platforms for presenting antigen epitopes. One of their advantages is that PNFs reveal high biocompatibility and did not promote tissue inflammation.^38,39^ In addition, enhanced nerve fiber growth from primary rodent neurons has been demonstrated on SAP matrices. In these cell culture experiments, SAPs enhanced axon re-growth and regeneration. So far, SAPs have mainly been used in central nervous system) CNS (injury models such as spinal cord lesions. These SAPs reduced glial scarring and neuro inflammation while simultaneously promoting outgrowth and functional recovery of severed axons. In contrast, there are only few studies of PNFs in peripheral nervous system (PNS) regeneration. So far, two model systems of PNS regeneration, i.e. facial^40^ or sciatic nerve regeneration were mainly employed. Different PNFs were used including nanofibers composed of C16VVVAAAEEE or RADA 16-I derived SAPs with two functional motifs IKVAV and RGD. These nanofibers were incorporated into collagen tubes, poly lactic-*co*-glycolic acid (PLGA) tubes or hydrogels.^41^ Short SAPs have been identified that formed PNFs capable of enhancing PNS regeneration without additional bioactive peptides, growth factors, or hormones. Bioactive SAPs were identified by screening a peptide library consisting of short peptide sequences with an average of nine amino acids per peptide that were stabilized by intermolecular ß-sheet structures. The facial nerve in mice to study PNS regeneration has been used. Facial motor neurons in the brain are connected by the facial nerve with several facial muscles to control e.g., whisker movement.

In other studies, on nerve regeneration, SAPs were introduced into hydrogels or tubes, which can lead to heterogeneous distribution and aggregation of the material. Dispersed SAP nanofibers in solution at the lesion site have been directly injected, where the SAPs revealed highly adhesive behavior and persisted at the lesion site for several weeks. The SAPs identified enhanced axon regeneration and they augmented adhesion and growth of primary PNS neurons.^41^

**Figure 13.**
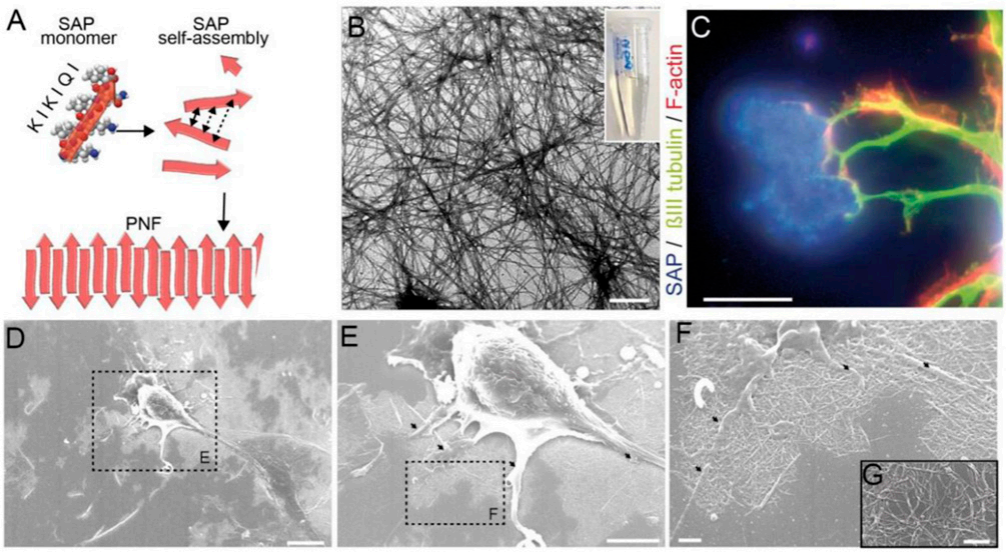
(A) Schematic illustration of SAP assembly forming PNFs. A SAP monomer consisting of the amino acid sequence KIKIQI interacts with other monomers to form PNFs. (B) TEM picture of PNFs based on SAP1e in solution. PNF are up to several 100 P.m. in length. (C) PNFs form plaques (blue) on glass coverslips serving as adhesion points for nerve fibers (green). (D-G) Scanning EM pictures of primary neurons plated on SAP1e PNFs. Neurons contact PNFs with several cellular protrusions (arrows in E, F) and PNFs are arranged in networks (F, G).^42^

In the other study, the SAPs were coated as two-dimensional matrix on glass coverslips to assess their ability to interact with PNS neurons. Adult mouse dorsal root ganglion (DRG) neurons have been used as well-established PNS neuron cell type.^42^ Figure 13(A) shows the schematic illustration of SAP assembly forming PNFs. A SAP monomer consisting of the amino acid sequence KIKIQI interacts with other monomers to form PNFs. Also, TEM picture of PNFs based on SAP1e in solution shows in Figure 13(B). In culture, neuronal cell bodies form several protrusions, so-called neurites that differentiate into either an axon or a dendrite. It has been observed that neurite tips, so-called growth cones, were frequently contacting SAP positive areas on the coverslip (Figure 13(C)). The spatial pattern of SAP localization on glass surfaces have been analyzed by scanning electron microscopy (Figure 13(D)–(F)). Typically, PNFs were arranged in networks (Figure 13(F) and (G)) forming plaques with areas up to 1 mm^2^ (Figure 13(D)). Neurons localized on top of these plaques (Figure 13(D)) contacted the PNFs with several protrusions (arrows in Figure 13(E) and (F)).^42^

**Figure 14.**
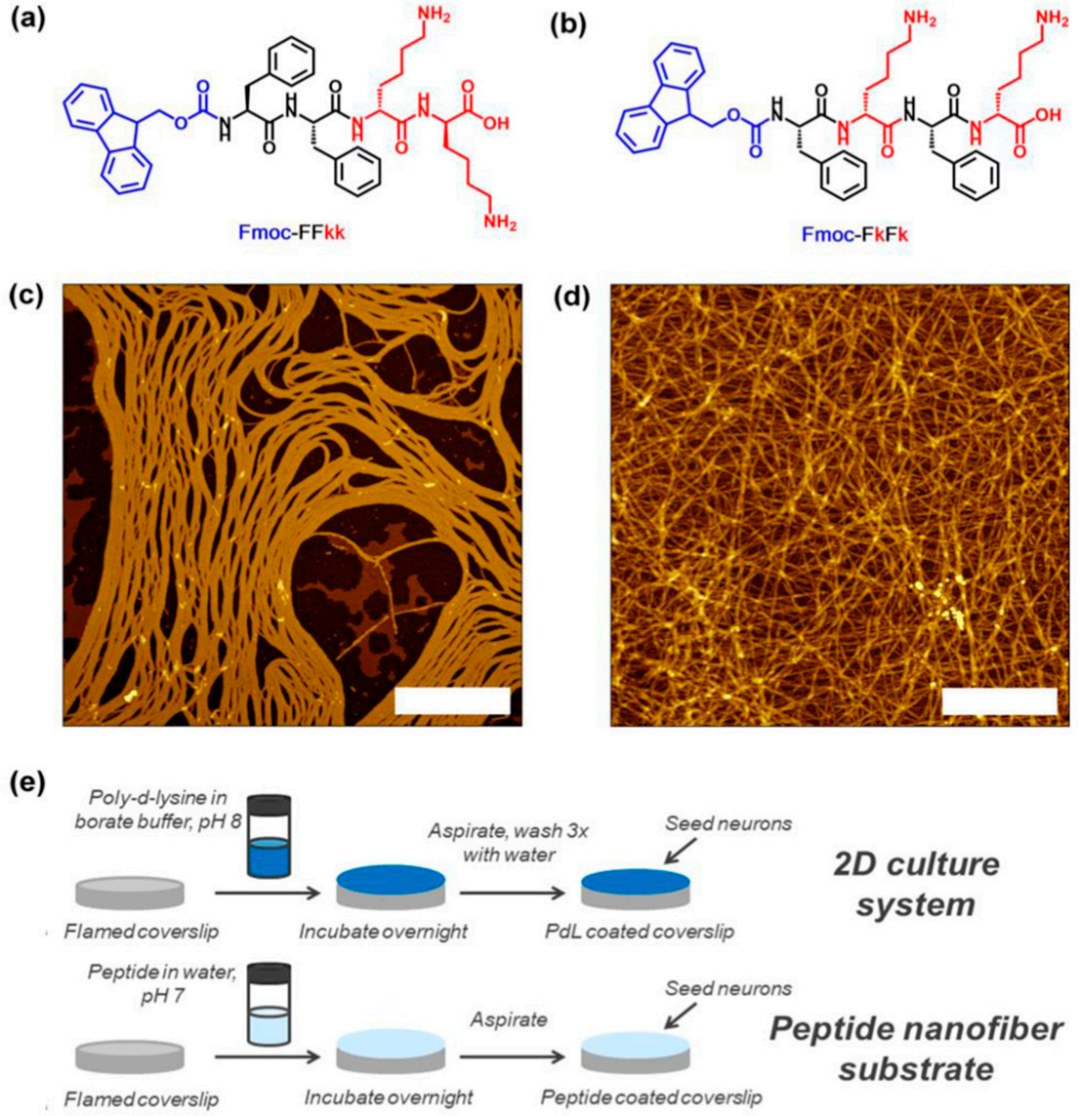
Lysine containing tetrapeptides (a) Fmoc-Phe-Phe-Lys-Lys and (b) Fmoc-Phe-Lys-Phe-Lys, (c and d) atomic force microscopy images of their self-assembly into fibers, and (e) the method employed to create peptide nanofiber coatings on glass coverslips, compared to traditionally used 2D cultures.^43^

In the other research, the two-novel tetra-peptides Fmoc-FFkk and Fmoc-FkFk, which self-assemble into fibers when dissolved in water has been developed (see Figure 14(a) and (b)). Peptides Fmoc-Phe-Phe-D-Lys-D-Lys (Fmoc-FFkk, where the lower-case k denotes the presence of a D-lysine) and Fmoc-Phe- D-Lys- Phe- D-Lys (Fmoc-FkFk), bearing two lysine groups. The use of chemically well-defined, self-assembling tetra-peptides as substrates for primary neuronal culture has been reported. These nanofibers can be coated atop glass coverslips, which were used to successfully culture primary neurons. Short peptides offer an alternative where the physical and chemical properties of the peptide can be tuned using a facile, scalable solid phase synthetic method. This includes charge, self-assembly kinetics and fiber morphology. Furthermore, self-assembled peptides are chemically uniform, reducing any batch-to-batch variability between substrates. These tetra peptides offer significant time savings over the widely used (poly-D-lysine) PDL, as no washing steps are required before seeding of primary neurons onto coverslips. Nanofiber coatings of Fmoc-FFkk and Fmoc-FkFk can be used to culture neurons long-term in vitro. Peptides Fmoc-Phe-Phe-D-Lys-D-Lys and Fmoc-Phe- D-Lys- Phe- D-Lys, bearing two lysine groups (Figure 14(a) and (b)) were dissolved in water and spontaneously self-assemble into fibers with β-sheet secondary structures, according to circular dichroism, viscosity, and atomic force microscopy measurements. AFM samples were prepared at a concentration of 0.5% (w/v) peptide and spread coated onto a freshly cleaved mica substrate (see Figure 14(c) and (d)). The method employed to create peptide nanofiber coatings on glass coverslips, compared to traditionally used 2D cultures has been shown in Figure 14(e).^43^

**Figure 15.**
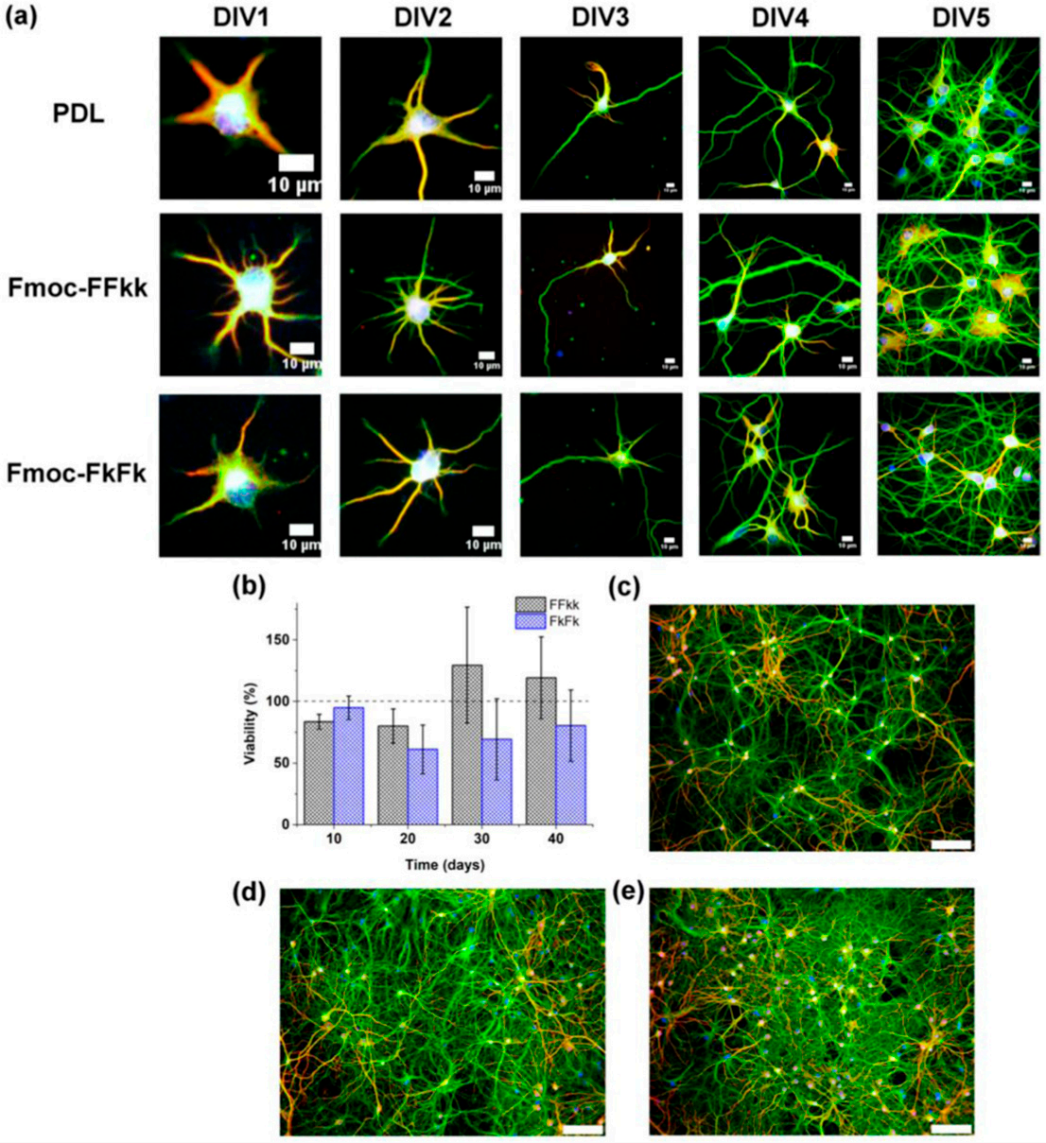
(a) Development of primary neurons seeded atop PDL or peptide nanofibers from 1 to 5 days in vitro (DIV). Long-term neuronal cultures on nanofiber substrates showing (b) cell viability of primary neurons seeded on peptide nanofibers as determined using an Alamar Blue colorimetric assay with PDL used as the positive control (100% viability). (c) Primary neurons fixed after DIV40 cultured on PDL, (d) Fmoc-FFkk, and (e) Fmoc-FkFk. In all cases, neurons are stained for β3-tubulin (green), MAP2 (red), and nucleus (blue).^43^

Development of primary neurons seeded atop PDL or peptide nanofibers from 1 to 5 days in vitro (DIV) has been shown in Figure 15. (Days In Vitro.” It refers to the number of days that cells have been cultured in a laboratory setting, typically on a culture dish or plate, as opposed to inside a living organism). Cultures were fixed and stained every 24 h for 1−5 days in vitro (DIV) (Figure 15(a)). No differences were observed in the early stages of neuronal development between Fmoc-FFkk, Fmoc-FkFk, and poly-D- lysine substrates. However, at longer time points, no significant differences in viability relative to PDL were observed (Figure 15(b)). And despite potential differences observed in the early stages of neuronal development between substrates, no significant discrepancies in viability were noted over extended culture periods compared to PDL. Staining of cultures fixed at DIV40 revealed that these long-term cultures form dense networks on peptide nanofiber substrates (Figure 15(d) and (e)), comparable to PDL (Figure 15(c)). This clearly indicates that Fmoc-FFkk and Fmoc-FkFk substrates support the long-term culture of primary neurons. The results suggested that the nanofiber substrates of Fmoc-FFkk and Fmoc-FkFk support the early stages of neuronal development. This clearly indicates that Fmoc-FFkk and Fmoc-FkFk substrates support the long-term culture of primary neurons also these substrates are permissive for neuronal growth, which do not invoke neuronal cell death or promote growth of non-neuronal cell types.

### Short laminin peptide for improved neural stem cell growth

**Figure 16.**
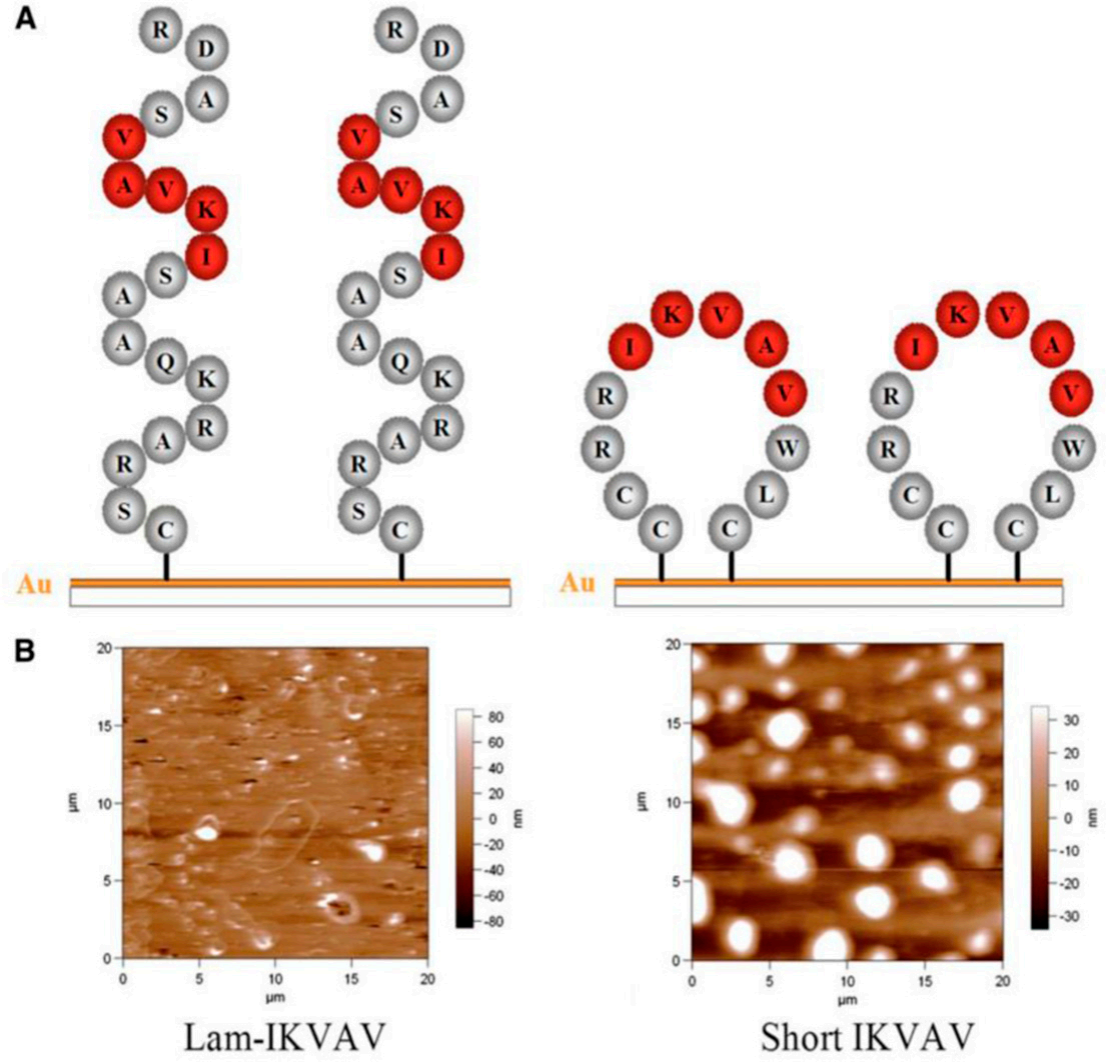
(A): Scheme of peptides. (B): Morphology of peptides inspected by atomic force microscope.^39^

**Figure 17.**
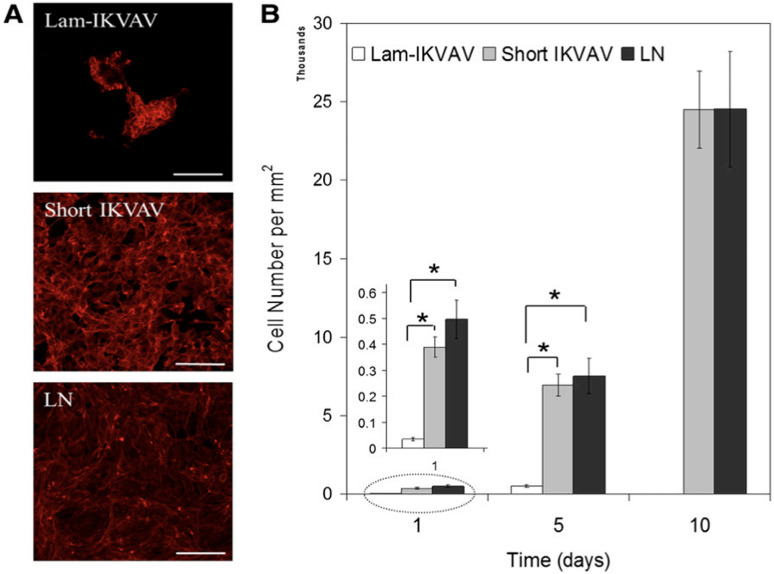
(A): Human neural stem/progenitor cells were stained with phalloidin (red). Scale bars = 100 mm. (B): Quantification of cell attachment and proliferation. Cells were assayed with CyQuant proliferation kit. A greater number of cells attached on cover slips conjugated with short IKVAV peptide than lam-IKVAV peptide at day 1.^39^

In this study, a novel peptide sequence has been developed with only 12 amino acids based on laminin sequence IKVAV: Ac-Cys-Cys-Arg-Arg-Ile-LysVal-Ala-Val-Trp-Leu-Cys (CCRRIKVAVWLC). This short peptide sequence, similar to tissue-derived full laminin molecules, supported hNSCs to attach and proliferate to confluence for continuous passage and subculture. This short peptide also directed hNSCs to differentiate into neurons. Morphology and AFM images of the lam-IKVAV peptide and short IKVAV conjugated to gold-coated cover slips has been shown in Figure 16. The novel peptide was examined for its ability to support the attachment, proliferation, and neuronal differentiation of hNSCs in two different contexts: coating on the two-dimensional (2D) substrates and conjugated to hydrogels based on polyethylene glycol (PEG) for three-dimensional (3D) culture. Morphology, attachment and proliferation of human neural stem/progenitor cells cultured on substrates coated with lam-IKVAV peptides, short IKVAV peptides, and whole LN has been shown in Figure 17. As shown in Figure 17, on the lam-IKVAV-coated surface, hNSCs preferred to aggregate together. In contrast, on the surface coated with shorter peptide, they spread more evenly, similar to those on whole-laminin-coated surfaces. On the 2D culture, its effects, relative to commercial laminin peptide Cys-Ser-Arg-AlaArg-Lys-Gln-Ala-Ala-Ser-Ile-Lys-Val-Ala-Val-Ser-Ala-Asp-Arg (CSRARKQAASIKVAVSADR; lam-IKVAV) has been compared and the whole protein laminin, on hNSC attachment, proliferation, and differentiation. When conjugated to PEG (Polyethylene Glycol)-based hydrogels, the effects of this short peptide on hNSC attachment, spreading, and proliferation on the surface of hydrogels as well as cell migration from human neuro spheres cultured inside hydrogels has been examined. When conjugated to poly (ethylene glycol) hydrogels, this short peptide benefited hNSC attachment and proliferation on the surface of hydrogels and promoted cell migration inside the hydrogels with maximum enhancement at a peptide density of 10 mM. This novel short peptide shows great promise in artificial niche development for supporting hNSC culture in vitro and in vivo and for promoting hNSC transplantation in future clinical therapy.^39^

### Bioactive self-assembling peptide hydrogels

**Figure 18.**
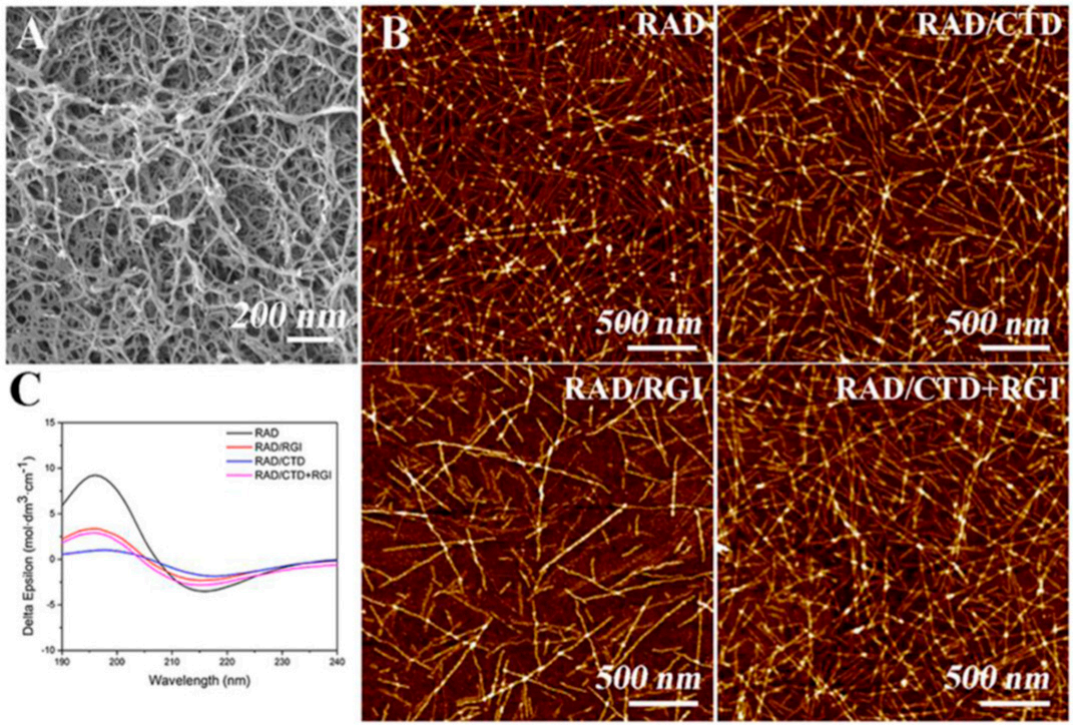
(A) Typical SEM morphology of self-assembling hydrogels. (B) AFM images of RADA16-I and solutions of various functionalized, self-assembling peptides mixed with RADA16-I. (C) CD spectra of RADA16-I and mixtures thereof with functionalized peptides, indicating that the mixtures had lower β-sheet levels than RADA16-I.^44^

In this study, a three-dimensional functionalized self-assembling peptide nanofiber hydrogel containing two neurotrophic peptides (CTDIKGKCTGACDGKQC and RGIDKRHWNSQ derived from nerve growth factor and brain-derived neurotrophic factor, respectively) has been prepared which reflected the structure and properties of the neural extracellular matrix. The material was used to promote axonal regrowth and functional recovery. Scanning electron microscopy revealed a three-dimensional porous matrix within the hydrogel. Circular dichroism spectroscopy and atomic force microscopy confirmed that the peptides displayed a β-sheet structure and self-assembled into long nanofibers. In this research, SEM (Figure 18(A)) and AFM (Figure 18(B)) confirmed that RADA16-I, RAD/RGI, RAD/CTD, and RAD/CTD + RGI featured self-assembled nanofibers that did not differ significantly on either the macro- or the microscale. RADA16-I (Ac-RADARADARADARADA-CONH2) is a typical (and widely used) self-assembling peptide exhibiting high-level biocompatibility, low cytotoxicity, a 3D structure facilitating cell growth, and good integration into variously shaped wounds. The material is composed of repeated segments containing positively charged arginine (R) and hydrophobic alanine (A) and negatively charged aspartic acid (D). RADA16-I facilitated peripheral nerve growth. Furthermore, various bioactive short-peptide motifs (analogs of growth factors and ECM macromolecules) are easily conjugated to the C-terminus of RADA16-I to balance the acidity of the solution and to promote specific cellular responses. RADA16-I hydrogel had the smallest fiber diameter, while RAD/CTD hydrogel showed the largest. There are more amino acids in CTDIKGKCTGACDGKQC than RGIDKRHWNSQ. The SEM showed that the hydrogels formed porous 3D matrices with pores 5–200 nm in diameter; the fiber diameters were about 10–30 nm, in correspondence with AFM results.^44^

## Control the self-assembly of conjugated molecules

**Figure 19.**
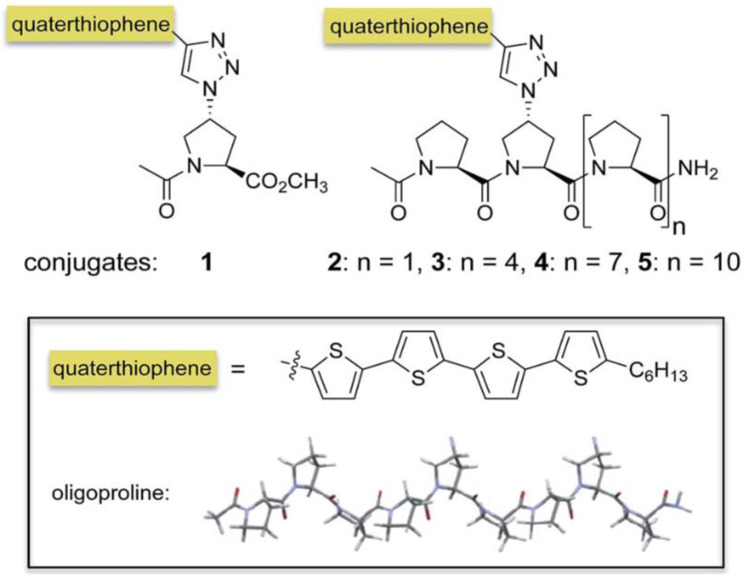
Oligoproline–quaterthiophene conjugates 1–5.^45^

Supramolecular assembly has become a powerful approach for the bottom-up formation of oligothiophene-based nanostructures in solution and their subsequent integration into devices. In particular, conjugates of oligothiophenes with biocompatible auxiliaries such as peptides or carbohydrates resulted in a variety of well-ordered, often chiral, self-assembled nanostructure. Oligoproline–quaterthiophene conjugates 1–5 have been shown in Figure 19. In this study, it has been shown that conjugation of a quaterthiophene moiety via a triazole linkage with oligoprolines allows for guiding the assembly of the chromophore. Variations of the length of the oligoproline moiety provided substantially different supramolecular assemblies, including nanostructured sheets, curls, or ribbons. It is shown that the length of the oligoproline affects the assembly of the quaterthiophenes into parallel or antiparallel π–π stacked aggregates and their further assembly into the higher ordered architectures.

**Figure 20.**
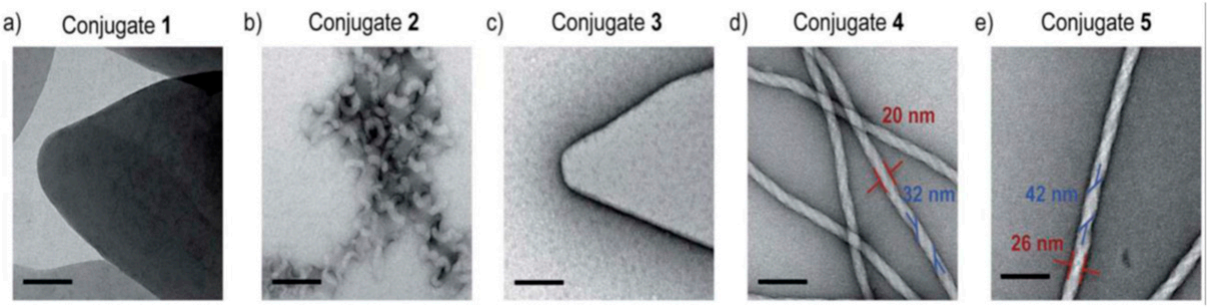
TEM micrographs of conjugates 1 (a), 2 (b), 3 (c), 4 (d), and 5 (e) deposited from THF: H_2_O 20:80 after annealing at 90 _C for 1 hour and cooling. Scale bars represent 100 nm.^45^

**Figure 21.**
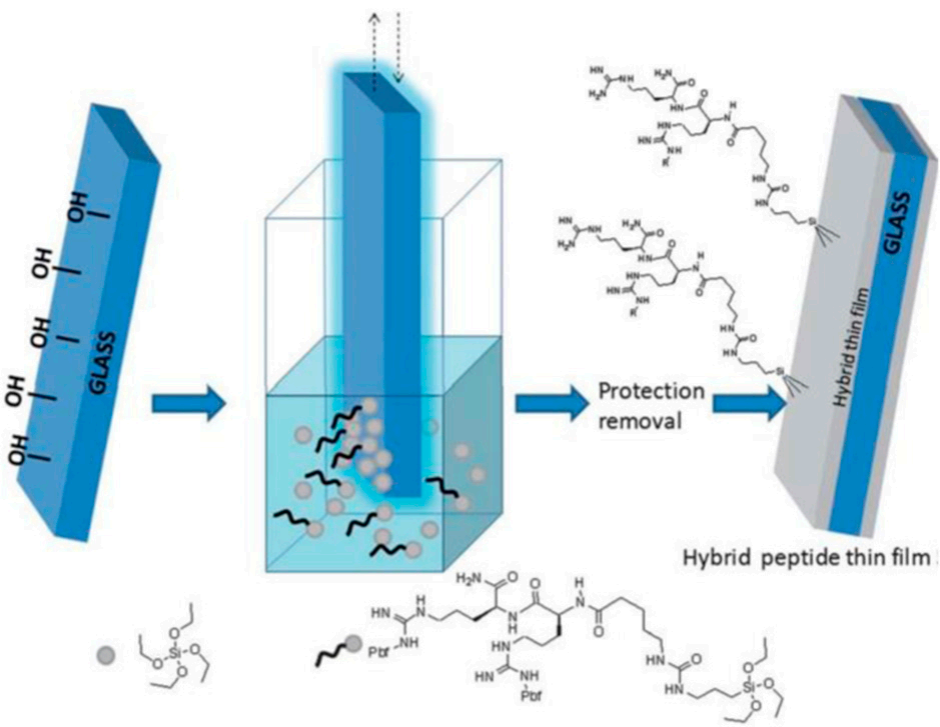
Direct synthesis of a hybrid peptide thin film by dip coating.^28^

It has been shown that oligoprolines, which do not self-assemble on their own, control the self-assembly of quaterthiophenes. The electron micrographs revealed distinct nanostructures dependent on the length of the oligoproline (Figure 20). TEM micrographs of self-assembled conjugate two show twirls that arise from tapes, which are curled up at the edges (Figure 20(b)). In contrast, the electron micrographs of nonamer four and dodecamer five furnish abundant nanofibrils consistently exceeding micrometer lengths with average widths of 20 nm and 26 nm, respectively (Figure 20(d) and (e), red). The TEM images also revealed that these fibrils are helical and have a right-handed twist. The average width of the twisted tape-like structures is 32 nm for 4 and 42 nm for 5, respectively (Figure 21(d) and (e), blue).^45^

## Cell adhesion and growth

**Figure 22.**
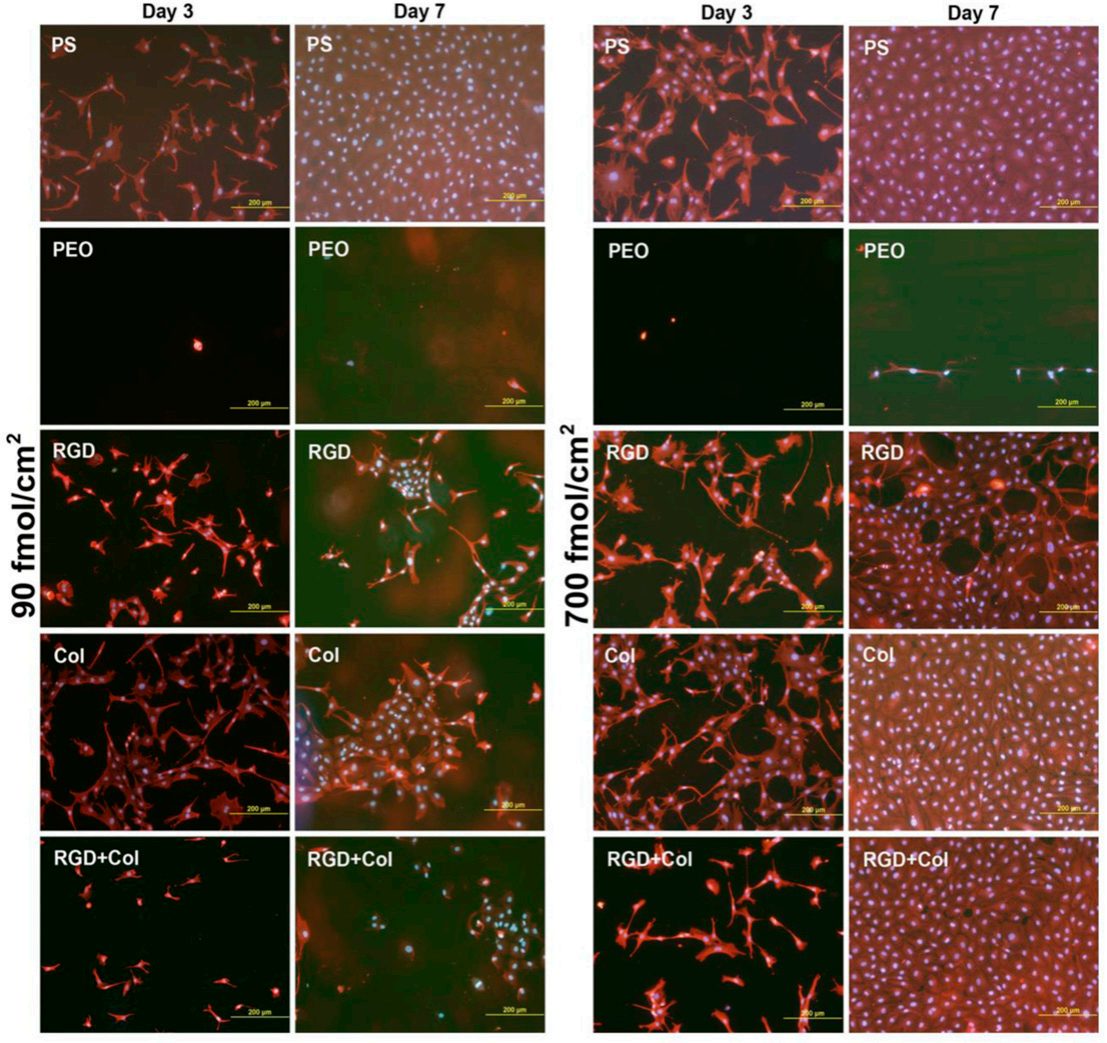
Morphology of endothelial CPAE cells on days 3 and 7 after seeding on standard cell culture polystyrene dishes (PS), on nonfouling PDA–PEO surfaces (PEO), and on PEO surfaces functionalized with RGD (RGD), with collagen-derived peptide (Col) or with a combination of RGD + Col at concentrations of 90 or 700 fmol/cm^2^ for each peptide.^46^

In this study, protein-repulsive polydopamine–poly (ethylene oxide) (PDA–PEO) surfaces were functionalized with an RGD-containing peptide (RGD), with a collagen-derived peptide binding fibronectin (Col), or by a combination of these peptides in concentrations of 90 fmol/cm^2^ and 700 fmol/cm^2^ for each peptide type. When seeded with vascular endothelial CPAE cells, the PDA–PEO surfaces proved to be completely non-adhesive for cells. On surfaces with lower peptide concentrations and from days 1 to 3 after seeding, cell adhesion and growth was restored practically only on the RGD-modified surface. Cell adhesion and growth was improved on surfaces modified with Col and with RGD + Col from days 3 to 7. On the other hand, the cells on the peptide-functionalized surfaces were more numerous, well-spread and mostly polygonal, although the number and spreading of cells on samples with the lower concentration of Col and RGD + Col were lower than on polystyrene and glass coverslips (Figure 22(a)). On samples with a higher concentration of Col and RGD + Col, the cell spreading area on day 3 after seeding was similar to that on control glass coverslips, and on day 7, the cells on these samples formed fully confluent layers similarly as on polystyrene (Figure 22). Only on surfaces with RGD, the cell spreading area was significantly smaller than on glass, and on day 7, these cells were subconfluent. At higher peptide concentrations, the cell adhesion and growth was markedly improved on all peptide-modified surfaces in both culture intervals. However, the collagen-derived peptide did not increase the expression of fibronectin in the cells. The deposition of fibronectin on the material surface was generally very low and similar on all peptide-modified surfaces. Nevertheless, the RGD + Col surfaces exhibited the highest cell adhesion stability under a dynamic load, which correlated with the highest expression of talin and vinculin in the cells on these surfaces. A combination of RGD + Col therefore seems to be the most promising for surface modification of biomaterials, e.g., vascular prostheses.^46^

## Multifunctional biomaterials for tissue engineering

**Figure 23.**
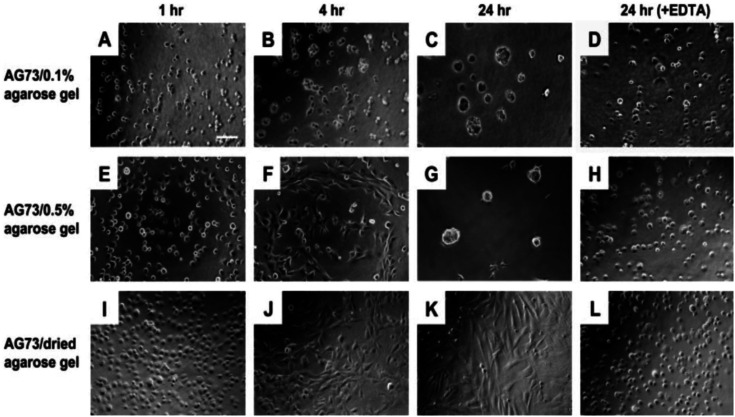
Human dermal fibroblasts morphologies on the AG73/agarose matrices. HDFs (4 × 104 cells/well) were cultured on the AG73 (30 pmol/mm^2^)/agarose matrices with serum-free media in the absence or presence of 5 mM EDTA for 24 h. After incubation, HDF morphologies were visualized using a BioZero microscope. (A–D) AG73/0.1% agarose gel; (E–H) AG73/0.5% agarose gel; (I–L) AG73/dried agarose gel. Scale bar = 100 μm. The AG73/dried agarose gels were prepared by drying of AG73/0.5% agarose gels.^47^

**Figure 24.**
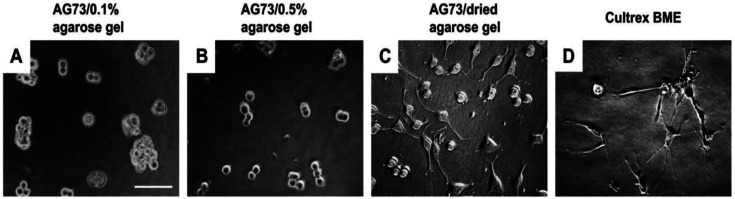
Neurite outgrowth on AG73/agarose matrices. Microphotographs were taken after 48 h. (A) AG73 (30 pmol/mm^2^)/0.1% agarose gel; (B) AG73 (30 pmol/mm^2^)/0.5% agarose gel; (C) AG73 (30 pmol/mm^2^)/dried agarose gel; (D) Cultrex BME. Scale bar ¼ 100 mm. The AG73/dried agarose gels were prepared by drying of AG73/0.5% agarose gels.^47^

In this study, functional polysaccharide matrices by mixing laminin active peptides and agarose gel has been prepared. Peptide AG73 (RKRLQVQLSIRT)/agarose matrices promoted strong cell attachment and the cell behavior depended on the stiffness of agarose matrices. In Figure 23, the human dermal fibroblasts morphologies on the AG73/agarose matrices has been shown. When human dermal fibroblasts (HDFs) were cultured on the AG73/dried agarose gel for 24 h, the cells strongly attached to the matrix and elongated after 4 h (Figure 23(I)–(K)). In contrast, HDFs formed spherical multicellular structures on the AG73/0.1% agarose gel (Figure 23(A)–(C)). On the AG73/0.5% agarose gel, cell spreading was observed at 4 h (Figure 23(F)). Then, the cells gradually formed multicellular spheroids and most cells formed the structures at 24 h (Figure 23(G)). Fibroblasts formed spheroid structures on the soft AG73/agarose matrices while the cells formed a monolayer with elongated morphologies on the stiff matrices. On the stiff AG73/agarose matrices, neuronal cells extended neuritic processes and endothelial cells formed capillary-like networks. Also, the effect of EDTA (ethylenediaminetetraacetic acid) on HDF morphological changes on the agarose matrices has been examined. Cell–cell adhesion and cell spreading were inhibited by EDTA (Figure 23(D), (H) and (L)). In the presence of EDTA, each cell showed a round shape, which is typical morphology for AG73-mediated cell attachment.^38^ PC12 cells swere cultured on the AG73 (30 pmol/mm^2^)/agarose matrices for 48 h. The AG73/dried agarose gel promoted neurite outgrowth (Figure 24(C)), although the activity was weaker than Cultrex BME (Figure 24(D)). Ratio of the cells with neurite on the AG73/dried agarose gel and Cultrex BME was 23.1 ± 4.1% and 94.7 ± 6.0%, respectively. On the other hand, no cells extended neurite on the AG73/0.1% and 0.5% agarose gels (Figure 24(A) and (B)). These results suggest that the stiff peptide/agarose matrices are suitable for neurite outgrowth as well as cell attachment, and demonstrate that bioactive peptides can provide not only cell attachment activity but also neurite outgrowth activity to the agarose gels.^48^

## Conclusion and outlook

Self-assembling peptides (SAPs), providing biomimetic synthetic micro-environments regulating cellular functionality and tissue repair, constitute a suitable tool for the production of complex tissue-like structures in vitro. Self-assembling peptides create fibrous hydrogels, which have been effectively tailored for a range of biomedical uses (refer to Table 1). In contrast to synthetic polymers that rely on chemical cross-linkers or covalent alterations, these peptides autonomously generate intricate three-dimensional fibrous hydrogels in physiological settings.

**Table 1.** Summary of representative self-assembling peptides.

Peptide	Motif/epitope conjugated	Application and reference
RADARADARADARADA	ALKRQGRTLYGF, DGRGDSVAYG, IKVAV, PGRDSGYRGDS, BMHP, YIGSR, RYVVLPR, TAGSCLRKFSTM, FHRRIKA, DGE, PFS, biotin	Cell scaffolds^49^
		Controlled release^50^
KLDLKLDLKLDL	KLPGWSG	Cell scaffolds^51^
FKFEFKFE	RGD	Controlled release^52^
FEFEFKFK		Drug delivery^53^
QQKFQFQFEQQ	RGDS, IKVAV, YIGSR	Cell scaffolds^54^
AEAEAKAKAEAKAKAK	His-tag	In situ antibody delivery^55^ and immobilization of target cells^56^

However, one of the most important drawbacks in 3D cultures, obtained via animal-derived substrates and serum-rich media, is the reproducibility and tunability of a standardized methodology capable to coax neural differentiation of different human cell lines. Creation of electronic devices based on SAP that enable the monitoring of cell-cell communication in respect to the cell surface morphology by controlling the deposition parameters, is very important. Even though the morphology of structures formed by different SAPs has been explored empirically; however, it remains an open challenge to understand the mechanistic relation between the peptide sequence in a SAPs and the resulting nanostructure. However, there have been few investigations on optimization peptide deposition parameters such as concentration, viscosity, temperature and speed in deposition process and the substrate also their relation to charge transport. Since the peptide assemblies show distinctive physicochemical and biochemical activities, depending on their morphology, size, and accessibility of the reactive surface area, in most cases, morphological control is the initial step in the design of functional peptide assemblies. Therefore, control over the molecular self-assembly by the processing and post-processing parameters and on the correlation between the resulting molecular organization and electrical properties in devices is very important. Peptide nanofiber can form networks resembling the extracellular matrix in key aspects that could serve as an artificial scaffold structure promoting cell adhesion and growth. We suggested the zone casting method for controlling the deposition condition because peptide deposition by zone-casing significantly improves the packing density and homogeneity of the peptide nanofiber film. The gained knowledge of efficient film processing of organic semiconductors to control their micro- and macrostructure organization will be transferred to the deposition of continuous thin films with well-defined surface morphology of biomolecules.

Zone-casting method allows an improvement of amyloid-fiber characteristics as well their organization on 2D platforms. Zone casting makes possible a continuous deposition of anisotropic layers of soluble materials. But it is important that for each compound, stationary deposition conditions must be determined by adjusting casting parameters such as temperature and casting speed. The unidirectional organization of peptides solution in zone-casting is achieved through careful control of mass transport, solution and substrate temperature, and substrate speed.^57–62^ Peptide fiber size remains constant as they are assembled in solution prior to deposition. Higher casting speeds lead to quicker solidification and thinner films due to increased cooling rates, whereas lower speeds result in thicker films by allowing the material to flow more freely. Faster solidification at higher speeds may result in higher defect densities, while slower speeds allow for better-ordered crystal structures and lower defect densities.^62–64^
